# Supplementary material for: Identification of photoperiod-induced specific miRNAs in the adrenal glands of Sunite sheep (Ovis aries)
Source: Front Vet Sci. 2022 Jul 22;9:888207. doi: 10.3389/fvets.2022.888207 (PMC9354845; doi:10.3389/fvets.2022.888207)
Supplement: Supplementary file 1 [file Data_Sheet_1.ZIP › Supplementary file/miRDeep2 file.zip!/miRDeep2 file/Novel_Structure/SP42A3/Novel_13.pdf]

Provisional ID	:	NC_019459.2_3582
Score total	:	2.4
Score for star read(s)	:	-1.3
Score for read counts	:	0
Score for mfe	:	2.1
Score for randfold	:	1.6
Score for cons. seed	:	
Total read count	:	76
Mature read count	:	74
Loop read count	:	0
Star read count	:	2

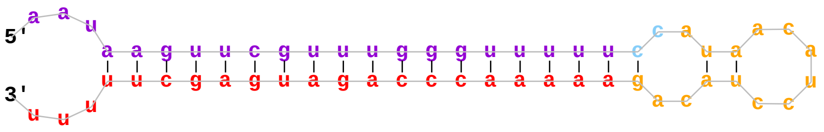

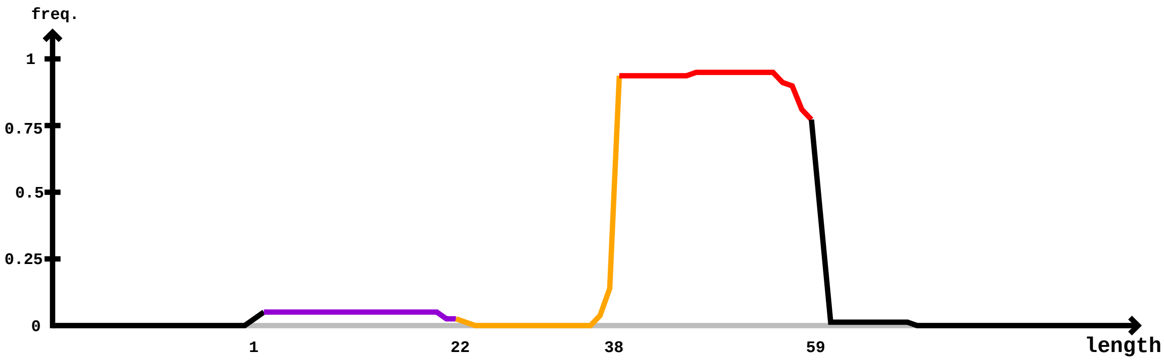


```
                          Star                                      Mature
5'- aaauacauguuggaguggccaauaaguucguuuggguuuuuccauaacauccuacagaaaaacccagaugagcuuuuuggccaacccaauacuuuauuuccuuuucggguc   -3'   obs
    aaauacauguuggaguggccaauaaguucguuuggguuuuuccauaacauccuacagaaaaacccagaugagcuuuuuggccaacccaauacuuuauuuccuuuucggguc         exp
    .......((((((..(((((((.(((((((((((((((((((..((......))..))))))))))))))))))).)))))))..))))))........(((....)))..   reads   mm   sample
    ...................caaAaaguucguuuggguuu........................................................................   2       1    seq
    ....................aauaaguucguuuggguuuuu......................................................................   2       0    seq
    .......................................................agaaaaacccagaugaAcuuuu..................................   3       1    seq
    ........................................................gaaaaacccagaugaAcu.....................................   1       1    seq
    ........................................................gaaaaacccagaugaAcuuu...................................   7       1    seq
    .........................................................aaaaacccagaugaAcu.....................................   2       1    seq
    .........................................................aaaaacccagaugaAcuu....................................   1       1    seq
    .........................................................aaaaacccagaugaAcuuuuu.................................   59      1    seq
    .........................................................aaaaacccagaugaAcuuuuug................................   1       1    seq
    .................................................................aAaugagcuuuuuggccaaccca.......................   1       1    seq
```
